# Identification and validation of matrix metalloproteinase hub genes as potential biomarkers for Skin Cutaneous Melanoma

**DOI:** 10.3389/fonc.2024.1471267

**Published:** 2024-10-18

**Authors:** Zhongyi Zhang, Mei Zhao, Zubing Zhou, Xiaodan Ren, Yunliang He, Tao Shen, Hongping Zeng, Kai Li, Yong Zhang

**Affiliations:** ^1^ School of Basic Medicine, Chengdu University of Traditional Chinese Medicine, Chengdu, China; ^2^ Institute of Traditional Chinese Medicine, Sichuan Academy of Chinese Medicine Sciences, Chengdu, Sichuan, China; ^3^ Department of Combined Chinese and Western Pulmonary Diseases, Zigong First People's Hospital, Zigong, Sichuan, China; ^4^ Sichuan Institute of Tourism College of Great Health Industry, Chengdu, Sichuan, China

**Keywords:** biomarker, diagnostic, MMPs, multi-scale methodology, SKCM

## Abstract

**Objectives:**

The role of matrix metalloproteinases (MMPs) in Skin Cutaneous Melanoma (SKCM) development and progression is unclear so far. This comprehensive study delved into the intricate role of MMPs in SKCM development and progression.

**Methods:**

RT-qPCR, bisulfite sequencing, and WES analyzed MMP gene expression, promoter methylation, and mutations in SKCM cell lines. TCGA datasets validated findings. DrugBank and molecular docking identified potential regulatory drugs, and cell line experiments confirmed the role of key MMP genes in tumorigenesis.

**Results:**

Our findings unveiled significant up-regulation of MMP9, MMP12, MMP14, and MMP16, coupled with hypomethylation of their promoters in SKCM cell lines, implicating their involvement in disease progression. Mutational analysis highlighted a low frequency of mutations in these genes, indicating less involvement of mutations in the expression regulatory mechanisms. Prognostic assessments showcased a significant correlation between elevated expression of these genes and poor overall survival (OS) in SKCM patients. Additionally, functional experiments involving gene silencing revealed a potential impact on cellular proliferation, further emphasizing the significance of MMP9, MMP12, MMP14, and MMP16 in SKCM pathobiology.

**Conclusion:**

This study identifies Estradiol and Calcitriol as potential drugs for modulating MMP expression in SKCM, highlighting MMP9, MMP12, MMP14, and MMP16 as key diagnostic and prognostic biomarkers.

## Introduction

Skin cutaneous melanoma (SKCM) is a malignancy arising from melanocytes, the pigment-producing cells located predominantly in the skin epidermis (1). SKCM is primarily caused by exposure to ultraviolet (UV) radiation from the sun or artificial sources like tanning beds (2). Prolonged exposure to UV radiation damages the DNA in skin cells, leading to mutations that can trigger melanoma development (3). Additionally, factors such as genetic predisposition, family history of melanoma, fair skin, presence of numerous moles, and weakened immune system also contribute to the risk of developing SKCM (4). Moreover, environmental factors, such as exposure to certain chemicals or radiation, and lifestyle habits like smoking, can further increase the likelihood of developing this aggressive form of skin cancer (5). Overall, a combination of genetic susceptibility and environmental exposures plays a significant role in the etiology of SKCM. SKCM represents an important global health burden due to its rising incidence rates and propensity for metastasis, accounting for the majority of skin cancer-related deaths worldwide (6). Despite advances in therapeutic strategies, including immunotherapy and targeted therapies, SKCM remains challenging to manage, emphasizing the urgent need for a deeper understanding of its molecular underpinnings to facilitate the development of effective diagnostic, prognostic, and therapeutic approaches.

Matrix metalloproteinases (MMPs) constitute a family of zinc-dependent endopeptidases that play pivotal roles in various physiological and pathological processes, including extracellular matrix (ECM) remodeling, tissue repair, inflammation, and cancer progression (7). Dysregulation of MMP expression and activity is implicated in the pathogenesis of numerous malignancies, where they mediate critical steps in tumor invasion, metastasis, angiogenesis, and immune evasion (8). MMPs facilitate tumor growth, invasion, and metastasis by remodeling the ECM and enabling cancer cells to migrate. MMPs are involved in various stages of cancer, from tumor initiation to metastasis (9). While MMPs have been extensively studied in multiple cancers, including breast, lung, colorectal, and prostate cancer, their roles in SKCM have garnered increasing attention in recent years.

In other cancers, MMPs have been implicated in various stages of tumorigenesis and cancer progression. For instance, MMP-2 and MMP-9 have been shown to promote tumor invasion and metastasis in breast cancer by degrading the ECM components and facilitating tumor cell migration (10, 11). Similarly, MMP-7 has been associated with enhanced invasiveness and metastatic potential in colorectal cancer, partly through its ability to cleave ECM proteins and promote epithelial-to-mesenchymal transition (EMT) (12). Moreover, MMP-14, also known as membrane-type 1 MMP (MT1-MMP), has been linked to tumor angiogenesis and metastasis in lung cancer by facilitating the degradation of basement membrane components and promoting the release of pro-angiogenic factors (13). In addition to their roles in tumor invasion and metastasis, MMPs have been implicated in modulating the tumor microenvironment (TME) to promote tumor growth and immune evasion (14). MMP-mediated ECM remodeling can release bioactive molecules sequestered within the ECM, such as growth factors and cytokines, thereby promoting tumor cell proliferation, survival, and angiogenesis (15). Moreover, MMPs can modulate the immune response by cleaving cell surface receptors, cytokines, and chemokines, thereby influencing immune cell trafficking, activation, and function within the TME (16).

Despite extensive research on matrix metalloproteinases (MMPs) in various cancers, their roles in SKCM remain unclear due to the cancer’s complexity and heterogeneity. MMPs are crucial for extracellular matrix remodeling, influencing tumor growth, invasion, and metastasis. Understanding their specific contributions to SKCM is essential for advancing diagnostic and therapeutic approaches. This study aims to bridge these gaps by integrating bioinformatics analyses and molecular experiments to evaluate the diagnostic, prognostic, and therapeutic implications of MMPs in SKCM. Identifying MMPs as potential diagnostic biomarkers could enhance early detection and patient stratification. Additionally, understanding their prognostic value could improve risk assessment and treatment personalization. The research also seeks to uncover novel therapeutic targets among MMPs, potentially leading to targeted therapies that could enhance treatment efficacy and patient outcomes. By utilizing multi-omics data, the study provides a comprehensive view of MMP functions in SKCM, offering new insights that could contribute to more effective and personalized treatment strategies.

## Methodology

**Figure 1:**
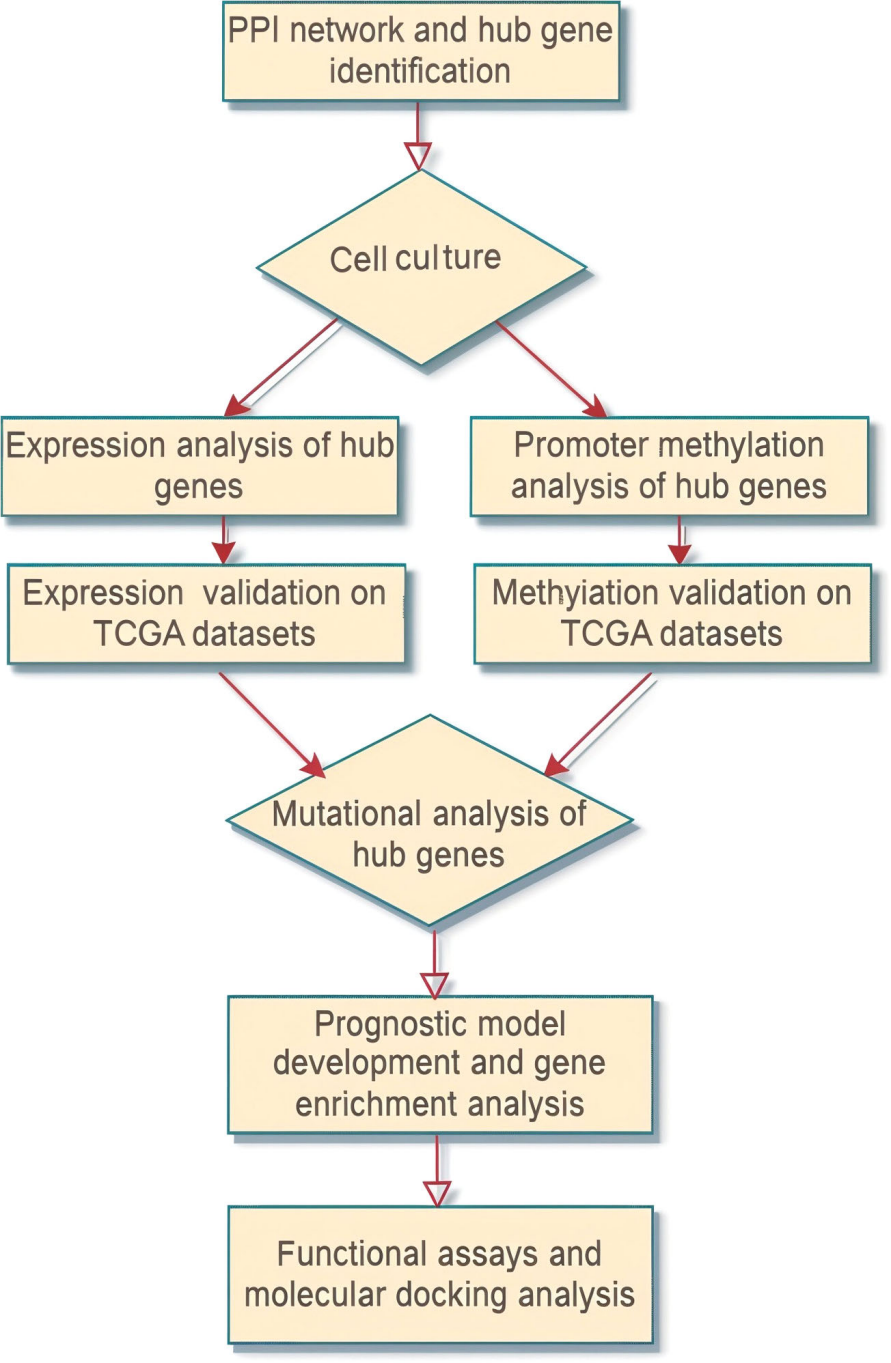
Flow sheet diagram depicting the methodology employed in the present study. The diagram provides a step-by-step visual outline of the experimental workflow.

The overall methodology of the present study is presented in **Figure 1**.

### List of the analyzed MMP genes in SKCM

In this investigation, a comprehensive analysis was conducted on a subset of 24 Matrix Metalloproteinase (MMP) family genes. The selected panel included MMP1, MMP2, MMP3, ILF3, MMP7, MMP8, MMP9, MMP10, MMP11, MMP12, MMP13, MMP14, MMP15, MMP16, MMP17, MMP19, MMP20, MMP21, MMP23B, MMP24, MMP25, MMP26, and MMP27. The primary objective was to identify and validate these genes as potential hub genes or molecular biomarkers with clinical relevance in SKCM patients.

### PPI construction and hub gene identification

The STRING database stands as a prominent and current resource for protein-protein interactions (PPIs), renowned for its comprehensiveness (17, 18). This database offers a seamlessly integrated platform that empowers researchers to delve into the intricate web of protein interactions and their roles in diverse biological systems, encompassing humans, yeast, bacteria, and more. This study harnessed the capabilities of the STRING web resource to construct the PPI network for the MMP protein family, adhering to the default settings.

Cytoscape software (19, 20) stands as a robust and widely employed tool, instrumental for researchers in scrutinizing protein-protein interaction networks. This software furnishes users with the means to visually represent the intricate tapestry of protein interactions and discern the pivotal participants within these networks. Leveraging its advanced algorithms and diverse plugins, Cytoscape enables tasks such as protein clustering, pathway analysis, and the creation of interactive visualizations, which prove invaluable in unraveling the intricacies of biological processes shaped by protein interactions. In this study, the Cytohubba plugin application (21) was employed with the Cytoscape platform to pinpoint hub genes within the constructed PPI network, utilizing the degree method. In more specific terms, the criteria for designating hub genes using the degree method involve ranking genes based on their connectivity, with those having the highest number of interactions being identified as hubs.

### Cell culture

In total, 20 SKCM cell lines (A2058, A375, WM793, SK-MEL-28, SK-MEL-2, G361, WM35, MeWo, HS294T, LOX IMVI, RPMI-7951, UACC-62, UACC-257, MALME-3M, HMCB, SK-MEL-5, SK-MEL-3, WM1552C, C32, IPC-298, and YUGEN8) as well as 20 normal skin cell lines (CCD-1106 KERTr, CCD-1112Sk, CCD-1121Sk, CCD-1140Sk, CCD-1152Sk, CCD-8Sk, Hs 895.Sk, Hs 936.Sk, Hs 919.Sk, Hs 888.Sk, Hs 895.T, Hs 27, Hs 852.T, Hs 895.C, Hs 895.O, Hs 895.P, Hs 895.R, Hs 895.D, Hs 895.B, and Hs 895.A), were procured from Pricella (Wuhan, Hubei, China) and subjected to STR matching analysis for verification. These cell lines were maintained under standard culture conditions at 37°C with 95% humidity and 5% CO_2_. The cell lines were cultured in DMEM (Dulbecco’s Modified Eagle Medium) supplemented with 10% fetal bovine serum (FBS), 1% penicillin-streptomycin, and 1% glutamine, ensuring optimal growth and viability.

### Nucleic acid extraction

DNA extraction was carried out using the organic method as described in reference (22, 23), utilizing the Phenol-Chloroform Isoamyl Alcohol (PCI) from Thermo Fisher Scientific (catalog number 15593031), while RNA was isolated using the TRIzol method according to the procedure detailed in reference (24, 25) utilizing the TRIzol Reagent from Invitrogen (catalog number 15596026).

### RT-qPCR-based expression analysis

The quality and purity of the isolated RNA were assessed utilizing an Agilent Bioanalyzer (Santa Clara, CA, USA). Subsequently, RNA was subjected to reverse transcription to synthesize complementary DNA (cDNA) with a ReverTra Ace^®^ qPCR RT Master Mix from TOYOBO, Shanghai, China (catalog number FSQ-301). Quantitative Real-Time Polymerase Chain Reaction (qRT-PCR) was conducted using SYBR Green PCR mix (Thermo Fisher Scientific, Waltham, USA) on an ABI 7900HT FAST Real-Time PCR System (Applied Biosystems, Foster City, CA, USA). To ensure normalization, Glyceraldehyde-3-phosphate dehydrogenase (GAPDH) was employed as an internal control, with normalization conducted by calculating the ΔCT values (CT of target gene - CT of GAPDH) to account for variations in RNA input and reverse transcription efficiency. Primer efficiency for GAPDH and hub genes was validated through standard curve analysis, ensuring that the amplification efficiency was between 90% and 110%. Relative mRNA expression levels were determined using the 2^−ΔΔCT^ method. A Student’s t-test was used to find differences between gene expression among SKCM and normal control groups, with a P-value < 0.05 considered significant. The following primers were purchased from the OriGene, USA Company for the amplification of GAPDH and hub genes.

GAPDH-F 5’-ACCCACTCCTCCACCTTTGAC-3’,

GAPDH-R 5’-CTGTTGCTGTAGCCAAATTCG-3’

MMP9-F: 5’-GCCACTACTGTGCCTTTGAGTC-3’

MMP9-R: 5’-CCCTCAGAGAATCGCCAGTACT-3’

MMP12-F: 5’-GATGCTGTCACTACCGTGGGAA-3’

MMP12-R: 5’-CAATGCCAGATGGCAAGGTTGG-3’

MMP14-F: 5’-CCTTGGACTGTCAGGAATGAGG-3’

MMP14-R: 5’-TTCTCCGTGTCCATCCACTGGT-3’

MMP16-F: 5’-GATTCAGCCATTTGGTGGGAGG-3’

MMP16-R: 5’-CCCTTTCCAGACTGTGATTGGC-3’

For GAPDH, the PCR conditions included an initial denaturation step at 95°C for 10 minutes, followed by 40 cycles of denaturation at 95°C for 15 seconds, annealing at 60°C for 30 seconds, and extension at 72°C for 30 seconds.

For MMP9, the PCR conditions were similarly set with an initial denaturation at 95°C for 10 minutes, followed by 40 cycles of denaturation at 95°C for 15 seconds, annealing at 59°C for 30 seconds, and extension at 72°C for 30 seconds.

For MMP12, the PCR conditions comprised an initial denaturation at 95°C for 10 minutes, followed by 40 cycles of denaturation at 95°C for 15 seconds, annealing at 54°C for 30 seconds, and extension at 72°C for 30 seconds.

For MMP14, the PCR conditions included an initial denaturation at 95°C for 10 minutes, followed by 40 cycles of denaturation at 95°C for 15 seconds, annealing at 51°C for 30 seconds, and extension at 72°C for 30 seconds.

For MMP16, the PCR conditions were set with an initial denaturation at 95°C for 10 minutes, followed by 40 cycles of denaturation at 95°C for 15 seconds, annealing at 58°C for 30 seconds, and extension at 72°C for 30 seconds.

The RT-qPCR assay and annealing temperatures of the primers were optimized using a serial dilution and gradient PCR methods to ensure accuracy and reliability. All reactions were performed in triplicates to ensure accuracy and reproducibility of the results.

### Receiver operating characteristic (ROC) curve

The Receiver Operating Characteristic (ROC) curve provides a holistic assessment, incorporating the continuous variables of sensitivity and specificity. The Area under the ROC curve (AUC) serves as an indicator of the diagnostic efficacy of the test. Typically, an AUC exceeding 0.9 is indicative of a highly accurate diagnostic test. The ROC curve analysis was carried out using Graph Pad Prism 7.0 with data derived from RT-qPCR and methylation analysis.

### Western blot analysis

Protein extracts from SKCM and normal control cell lines were resolved using 11% SDS-PAGE and subsequently transferred onto polyvinylidene difluoride (PVDF) membranes (Millipore). Following a 1-hour blocking step with 5% non-fat milk at room temperature, the PVDF membranes underwent three 10-minute washes with phosphate-buffered saline (PBS). Subsequently, the membranes were subjected to an overnight incubation at 4°C with primary antibodies targeting MMP9 (abcam, ab38898), MMP12 (abcam, ab137444), MMP14 (abcam, ab51074), MMP16 (abcam, ab73877), and control protein β-actin (abcam, ab8227), used as a loading control due to its stable and ubiquitous expression. After thorough washing, the membranes underwent a 2-hour incubation with secondary antibodies. After an additional three 10-minute washes with Tris-buffered saline/Tween-20 (TBST) at room temperature, the immunoreactivity was visualized using an ECL kit (Sangon Biotech), and the membranes were then exposed to Kodak XAR-5 film (Sigma-Aldrich).

### Promoter methylation analysis

#### Library preparation for targeted bisulfite sequencing analysis

1 µg of total DNA underwent fragmentation into 200-300 bp fragments using the Covarias sonication system (Covarias, Woburn, MA, USA). Following this, the DNA fragments underwent repair and phosphorylation of blunt ends, facilitated by a combination of enzymes including T4 DNA polymerase, Klenow Fragment, and T4 polynucleotide kinase. Subsequently, the repaired fragments underwent 3’ adenylation using Klenow Fragment (3’-5’ exo-), and then were ligated with adapters. These adapters featured 5’-methylcytosine instead of 5’-cytosine, along with index sequences, and the ligation was carried out using T4 DNA Ligase. After the library construction, quantification was conducted using a Qubit fluorometer with the Quant-iT dsDNA HS Assay Kit (Invitrogen, Carlsbad, CA, USA). Following this, the prepared libraries were sent to the Beijing Genomic Institute (BGI), China, for targeted bisulfite sequencing. Following the completion of sequencing, the methylation data underwent normalization, resulting in the generation of beta values.

### Mutational analysis

Mutations among the hub genes were explored through the Whole Exome Sequencing (WES) method. DNA from a total of 10 SKCM cell lines was sent to the Beijing Genomics Institute (BGI) and WES was performed according to the following protocol:

The targeted capture pulldown and exon-wide libraries were created from genomic DNA extracted from 10 SKCM cell line samples using the xGen^®^ Exome Research Panel from Integrated DNA Technologies, Inc., based in Illinois, USA, and the TruePrep DNA Library Prep Kit V2 for Illumina (#TD501, Vazyme, Nanjing, China). These captured libraries were subjected to pair-end sequencing on the Illumina HiSeq 2500 platform. Subsequently, the sequencing reads were processed and aligned to the GRCh37/hg19 human genome reference assembly, including the identification of germline variations. Local rearrangements were applied to enhance the alignment of individual reads. SNPs and insertion–deletion (indel) variants were called by implementing GATK’s Best Practices Workflow (for details, refer to https://github.com/Sydney-Informatics-Hub/Somatic-ShortV). Single nucleotide polymorphisms (SNPs) and insertion-deletion (indel) variants were identified by following the GATK’s Best Practices Workflow. This workflow involved the use of HaplotypeCaller to detect germline short variants and Mutech2 caller to identify somatic short variants, including SNVs and indels. For a comprehensive understanding of these procedures finally, the observed genetic mutations were interpreted according to the American College of Medical Genetics and Genomic (ACMG) guidelines (26) and annotated by utilizing the ClinVar database (27).

### Validation of hub expression using The Cancer Genome Atlas (TCGA) and Gene Expression Omnibus (GEO) datasets

Expression data from 472 SKCM samples in the TCGA database and 461 SKCM samples from the GEPIA (http://gepia.cancer-pku.cn/) (28) database were obtained. Additionally, data from 214 SKCM samples were extracted from the GSE65904 dataset available through the GEO database. The acquired data underwent normalization via log2 transformation using the normalized quantiles function from the preprocessCore package in R software. Subsequently, expression data for MMPs were filtered out to focus on relevant analyses.

### Validation of hub gene promoter methylation level and mutational analysis across The Cancer Genome Atlas (TCGA) datasets

MEXPRESS (https://mexpress.ugent.be/) stands as an invaluable resource catering to the needs of researchers and clinicians working in the field of oncology (29). This database serves as a repository of cancer-related information, encompassing critical details, including promoter methylation data. This study leveraged the capabilities MEXPRESS database to verify the promoter methylation status of the hub gene within the cohort of TCGA SKCM patients.

cBioPortal stands as a robust web-based platform, greatly simplifying the intricate task of delving into multifaceted cancer genomics data (30). This platform provides a user-friendly interface, empowering researchers to interactively dissect and visualize multifaceted cancer datasets, spanning genetic mutations and clinical information. In our study, the cBioPortal database was utilized for the mutational analysis of hub genes across the TCGA SKCM patients.

### Survival analysis and constriction of prognostic model

The KM plotter tool (https://kmplot.com/analysis/) serves as an indispensable asset for conducting survival analysis in the realm of cancer research (31). It furnishes researchers with an easily navigable platform to evaluate the influence of particular genes on patient survival. In the present research, the KM plotter tool was utilized to perform a survival analysis of the hub gene in SKCM patients.

To construct the prediction model, this study utilized the least absolute shrinkage and selection operator (Lasso) and multivariate Cox proportional hazard regression analysis, implemented using the “survival” package in the R language (32). The TCGA-ACC dataset served as the training dataset, while the GSE33371, GSE19750, and GSE10927 datasets were designated as validation datasets. In this analysis, positive coefficients indicated an increased risk of an event, such as death, while negative coefficients suggested a reduced risk. The magnitude of these coefficients reflected the impact of variables on hazard rates, which was instrumental in developing prognostic models for survival outcomes.

The formula for the prognostic model for SKCM patients’ prognosis was derived as follows:

Risk score = Σ (multivariate Cox regression coefficient variation of each mRNA). This formula allowed for the calculation of a risk score based on the sum of the multivariate Cox regression coefficient variations associated with each mRNA, thereby facilitating the prediction of prognosis for SKCM patients.

### Gene enrichment analysis

The present study performed gene enrichment analysis using the DAVID tool (https://david.ncifcrf.gov/) (33) on the identified hub genes. DAVID, a bioinformatics application, simplifies the functional analysis of extensive gene lists. Researchers can extract valuable insights into gene functions, pathways, and biological processes, enhancing their ability to interpret high-throughput genomics data.

### Exploration of hub expression regulatory drugs

DrugBank (https://go.drugbank.com/) is a prominent resource for comprehensive information on drugs, including their interactions, mechanisms of action, and therapeutic applications (34). This study harnessed the capabilities of DrugBank to investigate drugs that may regulate the expression of hub genes in the treatment of SKCM.

### Knockdown of hub genes in SKCM cell line

The siRNA designed to target hub genes was procured from OBiO Company. To knockdown hub genes (MMP9, MMP12, MMP14, and MMP16), melanoma cell lines were transfected with siRNA using a Transfection Reagent (INTERFERin, French). Following siRNAs were used to knockdown hub genes:

siMMP9 (Sense): 5’-CUAUGGUCCUCGCCCUGAATT-3’

siMMP9 (Anti-sense): 5’-UUCAGGGGCGACCAUAGTT-3’

siMMP12 (Sense): 5′-GCUGUUUUUAACCCACGUUTT-3′

siMMP12 (Anti-sense): 5′-CCGUGAGGAUGUUGACUACTT-3′

siMMP14 (Sense): 5′-AACAGGCAAAGCUGAUGCAGAdTdT‐3′

siMMP14 (Anti-sense): 5′-AAUCUGCAUCAGCUUUGCCUGdTdT‐3′

siMMP16 (Sense): 5′-CGUGAUGUGGAUAUAACCATT-3′

siMMP16 (Anti-sense): 5′-UGGUUAUAUCCACAUCACGTT-3′

Moreover, the knockdown efficacy of the MMP9, MMP12, MMP14, and MMP16 was assessed using RT-qPCR and western blot analyses following the previously mentioned protocols.

### Cell counting Kit-8 and colony formation assays

To assess the cell proliferation ability of melanoma cells, this study employed the Cell Counting Kit-8 (CCK-8) from APExBIO, USA. Initially, 3 × 10^3 cells per well were seeded into 96-well plates 24 hours post-transfection. Following an incubation period at 37°C for various durations (0, 24, 48, and 72 hours), CCK-8 reagent was added to each well, and the absorbance was measured at 450 nm. For the colony formation assay, 5 × 10^2 melanoma cells were cultured in 6-well plates for 10 days under conditions of 37°C and 5% CO_2_. Subsequently, the cells were stained with 0.1% crystal violet for 15 minutes, after which colony quantification was performed using ImageJ software. This approach allowed us to evaluate the proliferative capacity of melanoma cells over time and their ability to form colonies, providing valuable insights into their growth behavior and potential therapeutic targets.

### Molecular docking analysis

#### Ligand and receptor preparation and docking analysis

To evaluate the binding affinities between Estradiol, Calcitriol, and the MMP9, MMP12, MMP14, and MMP16 proteins, molecular docking analysis was conducted using the CB-DOCK (http://clab.labshare.cn/cb-dock/) web server (35). Estradiol and Calcitriol structures in SDF format were retrieved from the PubChem database (https://pubchem.ncbi.nlm.nih.gov/), while PDB structures for MMP9, MMP12, MMP14, and MMP16 proteins were generated using the SwissModel tool (https://swissmodel.expasy.org/). The process involved several crucial steps. Initially, ligand pre-processing was performed, followed by the removal of excess ligands from the target proteins and the elimination of crystal water molecules. Hydrogen atoms were then added to facilitate the molecular docking process. Subsequently, molecular docking was conducted using the CB-DOCK platform to compute the binding energies of the molecules across various conformations. Binding energies falling within the range of -5 kcal/mol to -10 kcal/mol or lower were considered favorable. The conformation with the highest hydrogen bond energy was identified as the active component of the protein interaction.

For visualization purposes, PYMOL software (version 2.5.2) was utilized to render the molecular interactions, enabling a comprehensive understanding of the binding modes and potential binding sites between the ligands (Estradiol, Calcitriol) and the target proteins (MMP9, MMP12, MMP14, MMP16). This approach allowed us to elucidate the molecular mechanisms underlying the interaction between the chosen drugs and the MMP proteins, providing valuable insights for further drug development and therapeutic intervention strategies.

### Statistics

In this study, Gene Ontology (GO) and Kyoto Encyclopedia of Genes and Genomes (KEGG) enrichment analyses were conducted to elucidate the biological pathways and functions associated with the studied genes. Fisher’s Exact test was utilized to compute the differences in enrichment between different gene sets (36). Additionally, correlational analyses were performed using the Pearson method to explore potential relationships between variables of interest. Furthermore, comparisons between groups were made using a Student’s t-test to assess statistical significance. A P-value < 0.05 was considered significant.

All statistical analyses were carried out using R version 3.6.3 software, a widely used and powerful tool for data analysis and visualization.

## Results

### PPI construction and identification of hub genes

**Figure 2:**
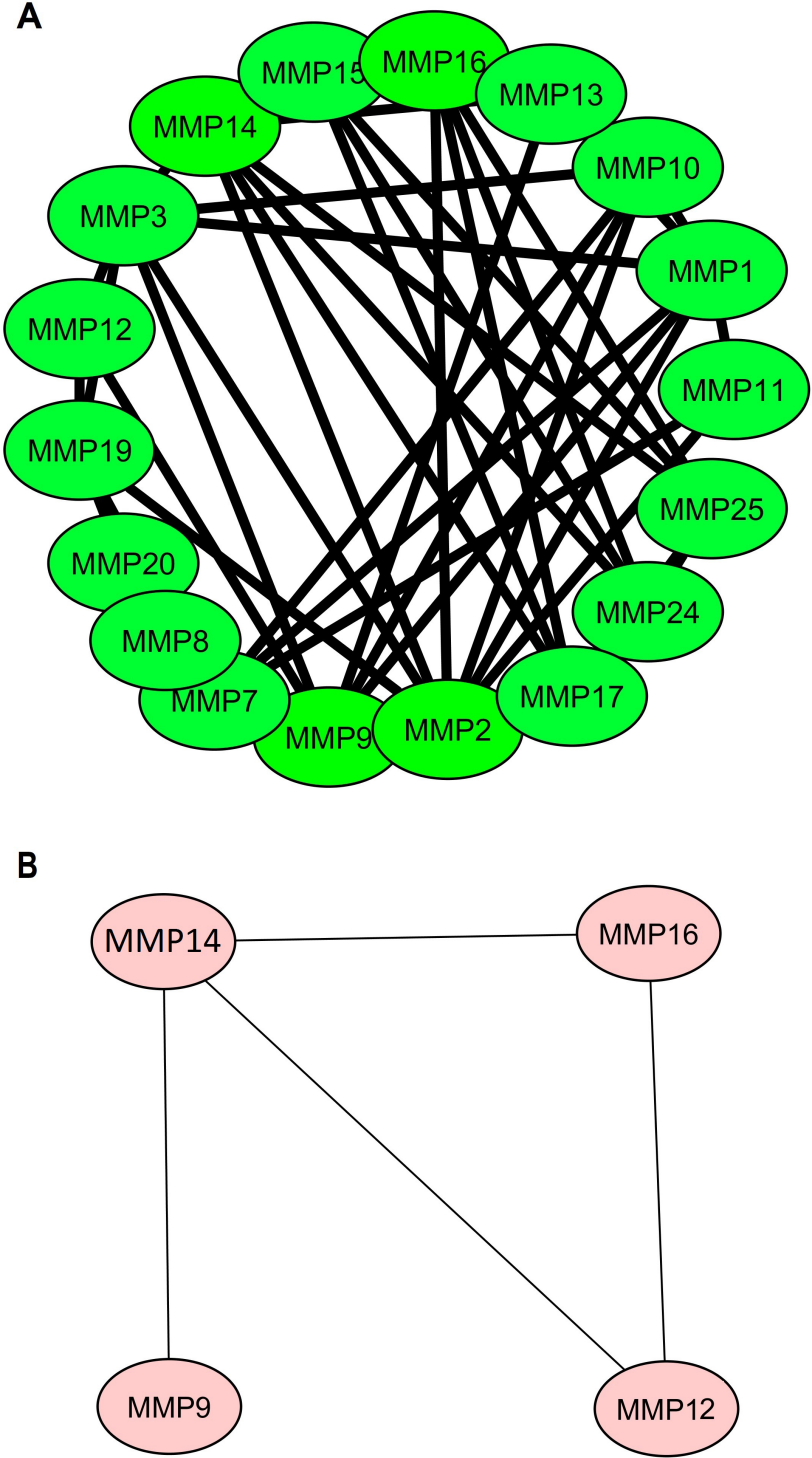
Protein-protein interaction (PPI) networks illustrating MMP family proteins and identified hub genes. **(A)** PPI network featuring MMP family proteins, and **(B)** PPI network focuses on the four identified hub genes (MMP9, MMP12, MMP14, and MMP16), which were highlighted based on centrality metrics in network analysis.

Firstly, a PPI network of the 24 MMP family members was established using the STRING web server (**Figure 2A**). Subsequently, this network was imported into Cytoscape software to identify hub genes using the degree method. The Cytohubba application within Cytoscape identified MMP9, MMP12, MMP14, and MMP16 as the hub genes (**Figure 2B**) with the highest degree of centrality.

### Experimental expression and promoter methylation analyses of hub genes in SKCM cell lines

**Figure 3:**
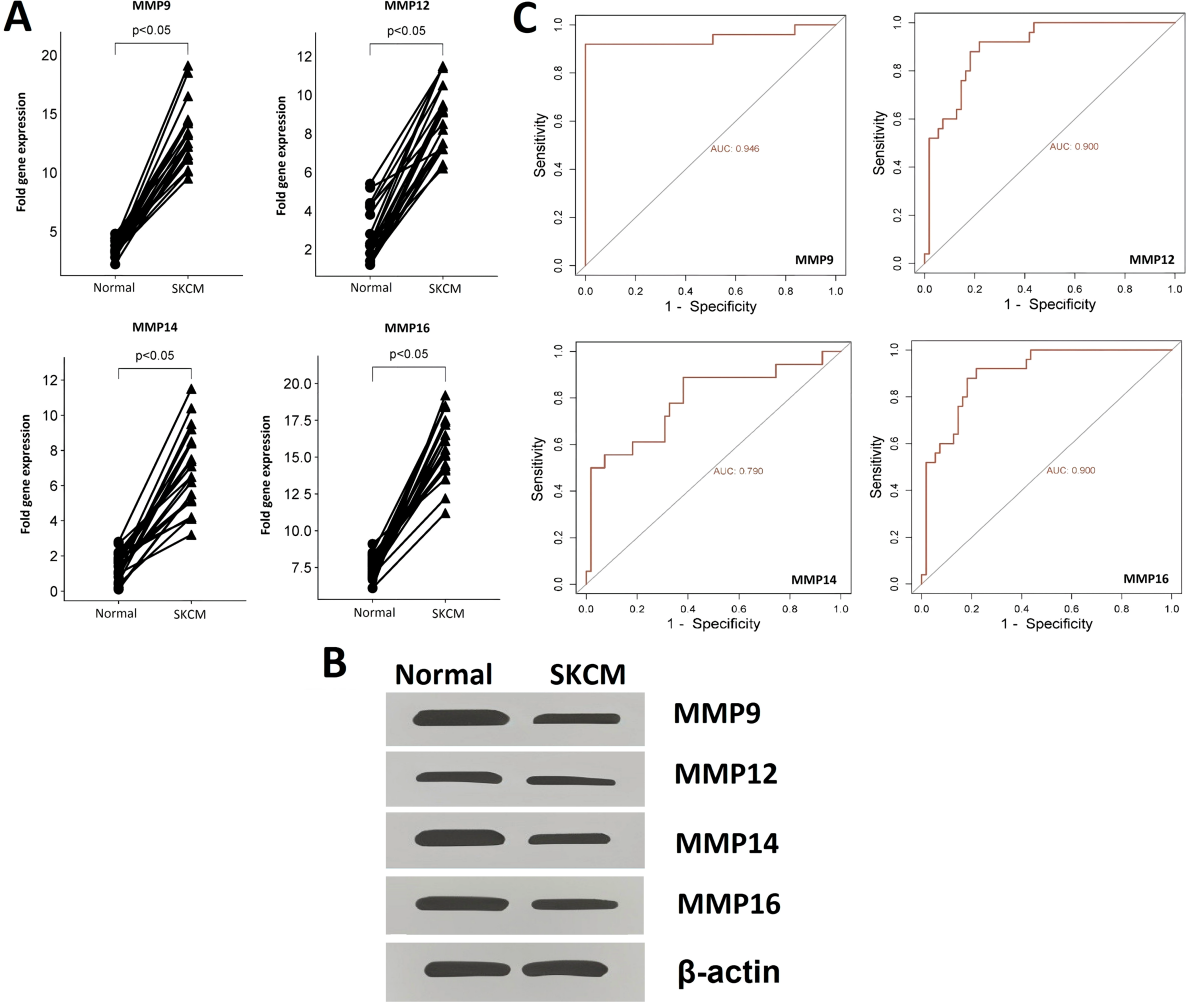
RT-qPCR and western blot-based expression profiling and ROC analysis of the hub genes **(A)** RT-qPCR-based relative expression of the hub genes in SKCM and normal control cell lines, **(B)** Western blot analysis-based expression of hub genes in SKCM and normal control cell lines, and **(C)** RT-qPCR expression-based ROC analysis of the hub genes. A p-value < 0.05 was considered significant.

The expression and promoter methylation levels of the hub genes across the SKCM (n = 20) and the normal control (n = 20) cell lines were compared through RT-qPCR, western blot, and bisulfite sequencing analyses. RT-qPCR analysis results revealed that across the SKCM cell lines, the mRNA expression of four hub genes (MMP9, MMP12, MMP14, and MMP16) was significantly higher as compared to the normal control cell lines (**Figure 3A**). Western blot analysis showed that protein expression of MMP9, MMP12, MMP14, and MMP16 was also higher in the SKCM cell lines group as compared to the normal controls (**Figure 3B**).

**Figure 4:**
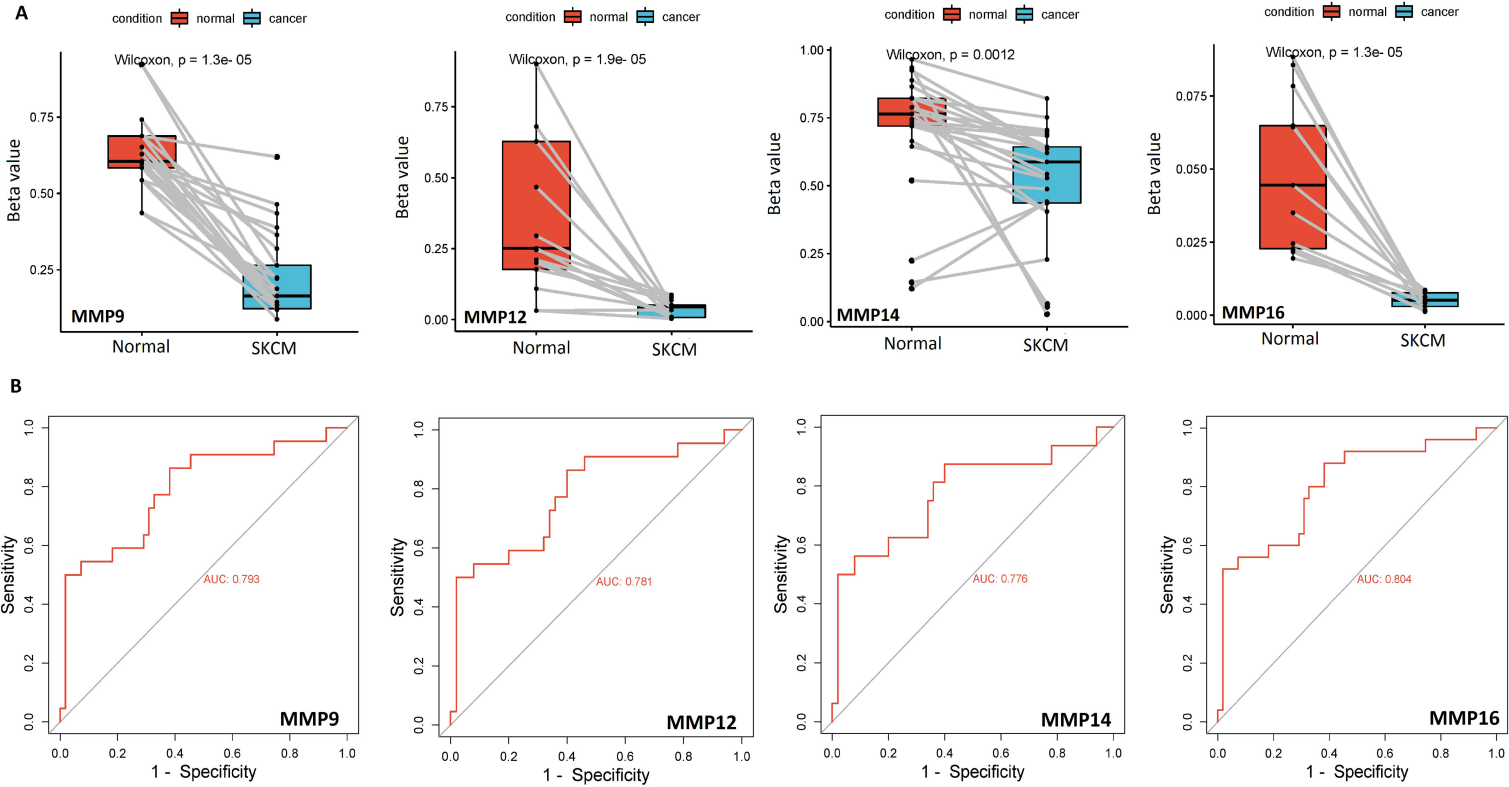
Bisulfite sequencing-based promoter methylation level profiling and ROC analysis of the hub genes **(A)** Bisulfite sequencing-based relative promoter methylation levels of the hub genes in SKCM and normal control cell lines, and **(B)** Promoter methylation level-based ROC analysis of the hub genes. A p-value < 0.05 was considered significant.

Additionally, the bisulfite sequencing outcomes indicated that the promoters of MMP9, MMP12, MMP14, and MMP16 genes exhibited lower methylation levels in the SKCM cell lines group when compared to the normal control cell line group, as illustrated in **Figure 4A**.

ROC analysis was conducted to evaluate the diagnostic potential of MMP9, MMP12, MMP14, and MMP16 expression, as well as promoter methylation, in SKCM patients. The observed AUC of > 0.775 suggests that MMP9, MMP12, MMP14, and MMP16 mRNA expression and promoter methylation levels have strong diagnostic accuracy for SKCM detection (**Figures 3C**, **4B**).

### Experimental mutational analysis of the hub genes across SKCM cell lines

Mutational analysis of the hub genes was conducted utilizing the WES technique in 10 SKCM cell lines. The results of this comprehensive analysis revealed that only one benign mutation (NM_004994.3 (MMP9):c.70C>T (p.Arg24Cys)) was identified in the MMP9 gene across three SKCM cell line samples. However, for the MMP12, MMP14, and MMP16 genes, no mutations were observed in the SKCM cell lines. These findings collectively underscore the infrequent occurrence of mutations in MMP9, MMP12, MMP14, and MMP16 genes within SKCM cell lines, suggesting that they are not commonly mutated in this context.

### Validation of hub expression using The Cancer Genome Atlas (TCGA) and Gene Expression Omnibus (GEO) datasets

**Figure 5:**
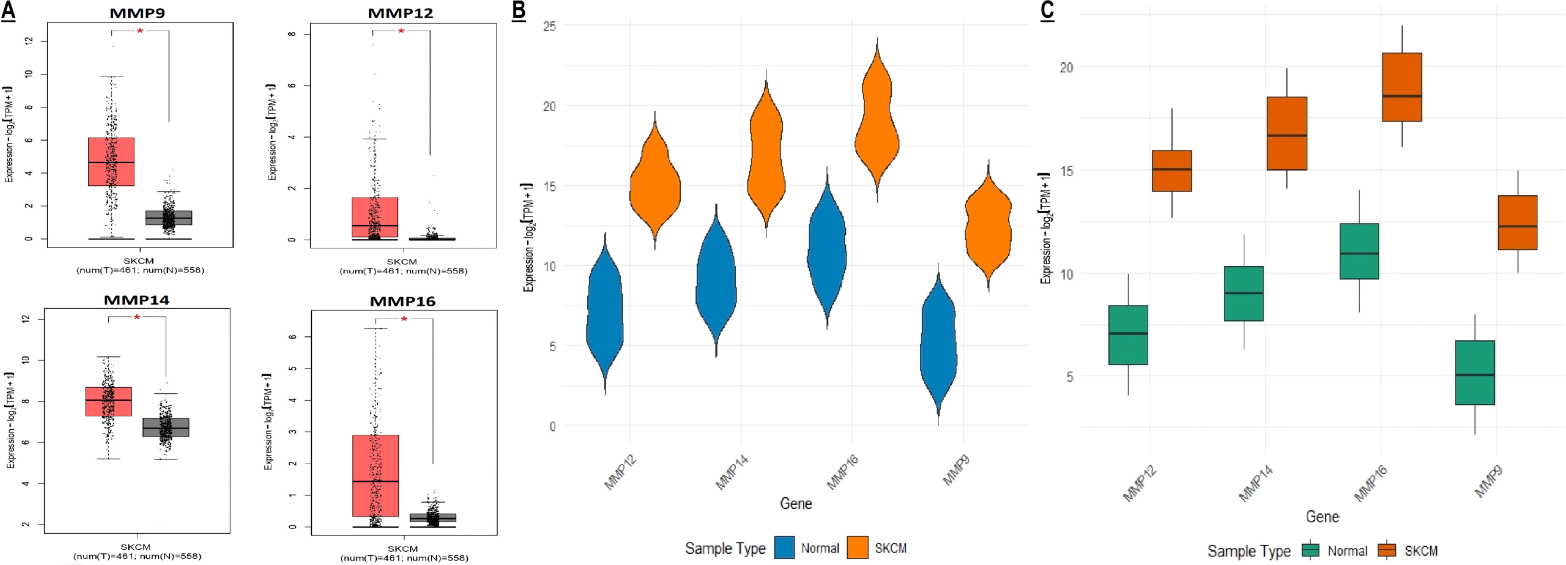
mRNA expression profiling of the hub genes using TCGA and GEO SKCM datasets. **(A, B)** Box plot presentation of hub gene mRNA expression in TCGA SKCM datasets, and **(C)** Box plot presentation of hub gene mRNA expression in GEO SKCM dataset (GSE65904). A p-value < 0.05 was considered significant. p*-value < 0.05.

In this part of the study, MMP9, MMP12, MMP14, and MMP16 mRNA expression in SKCM patients from TCGA project was validated from the GEPIA, TCGA, and GEO databases. Results of the 3 different SKCM datasets showed that the level of MMP9, MMP12, MMP14, and MMP16 mRNA expression was significantly higher in SKCM tissues as compared to the normal tissues (**Figures 5A–C**).

### Validation of hub gene promoter methylation level and mutational analysis across The Cancer Genome Atlas (TCGA) datasets

**Figure 6:**
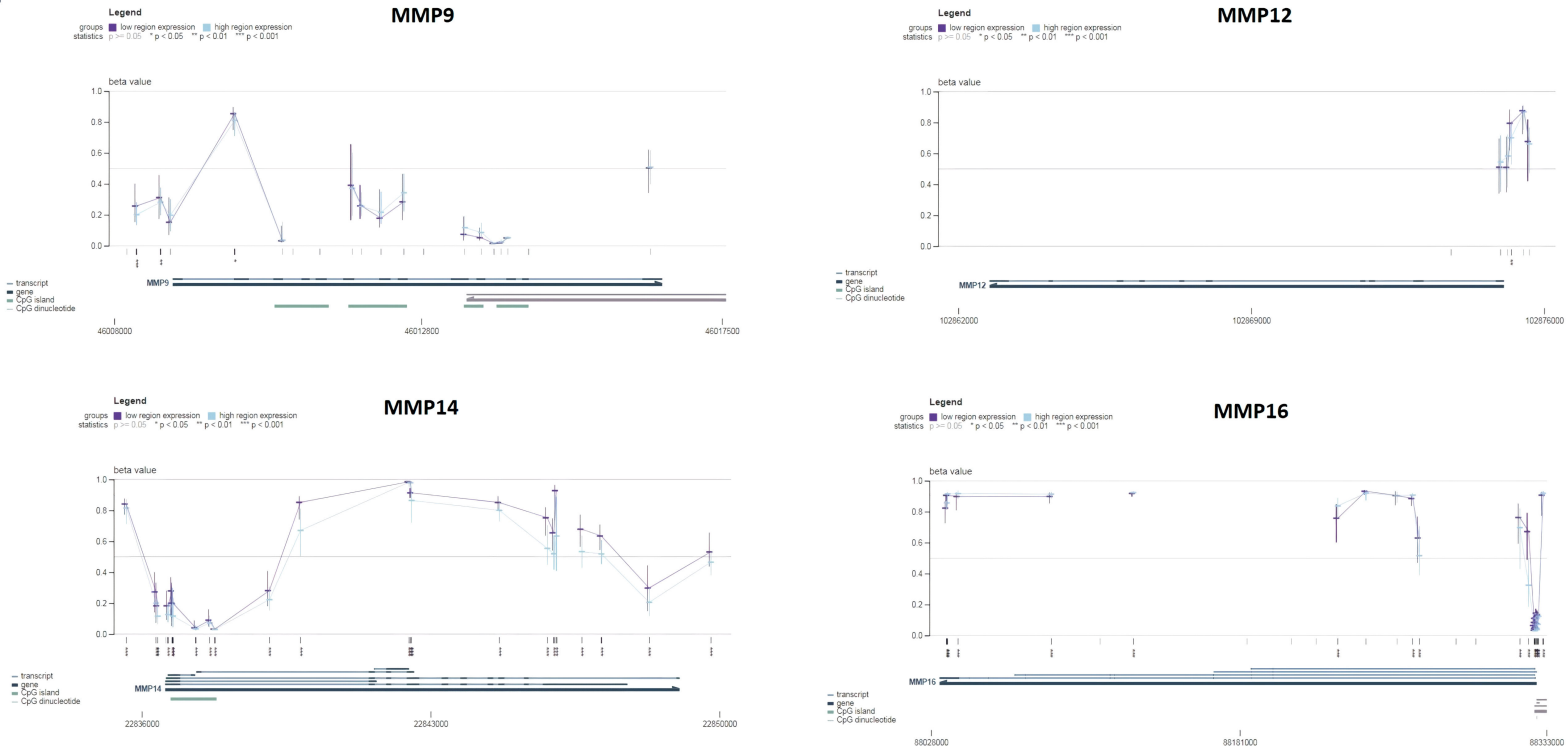
Promoter methylation analysis of the hub genes across TCGA SKCM and normal control samples via MEXPRESS database. This analysis provides insights into the epigenetic regulation of the hub genes in SKCM. A p-value < 0.05 was considered significant.

Next, the promoter methylation levels of the MMP9, MMP12, MMP14, and MMP16 genes were validated using the MEXPRESS. It was observed that the promoter methylation levels of the MMP9, MMP12, MMP14, and MMP16 were lower in the SKCM samples from TCGA relative to the corresponding controls (**Figure 6**). Taken together, these results indicate that decreased methylation levels in the promoters of MMP9, MMP12, MMP14, and MMP16 may be a contributing factor to the elevated expression of these genes in SKCM.

**Figure 7:**
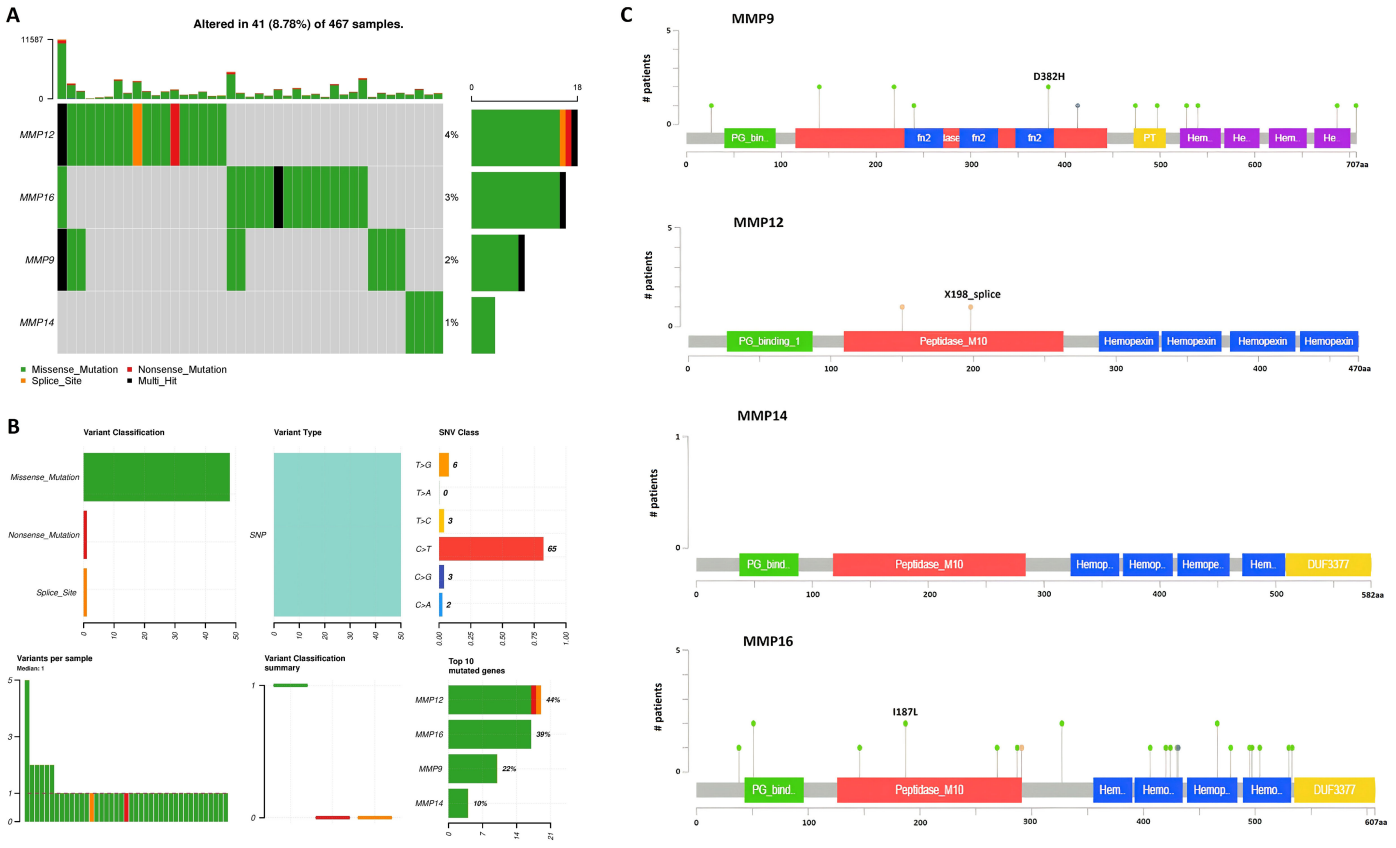
Mutational analysis of hub genes across TCGA SKCM samples via cBioPortal databases. **(A)** Percentage of the mutated SKCM samples, **(B)** Summery of the observed genetic alterations in hub genes across SKCM samples, and **(C)** depiction of amino acid change due to mutations at the protein levels.

To determine mutations in the MMP9, MMP12, MMP14, and MMP16 genes across TCGA SKCM samples, a comparative analysis of these genes was conducted using cBioPortal. The analysis revealed mutations in the hub genes MMP9, MMP12, MMP14, and MMP16 in only a small fraction (2%, 4%, 1%, and 3%, respectively) of the SKCM samples under investigation, suggesting that these mutations play a limited role in the aberrant regulation of these genes (**Figure 7**).

### Survival analysis and constriction of hub gene-based prognostic model

**Figure 8:**
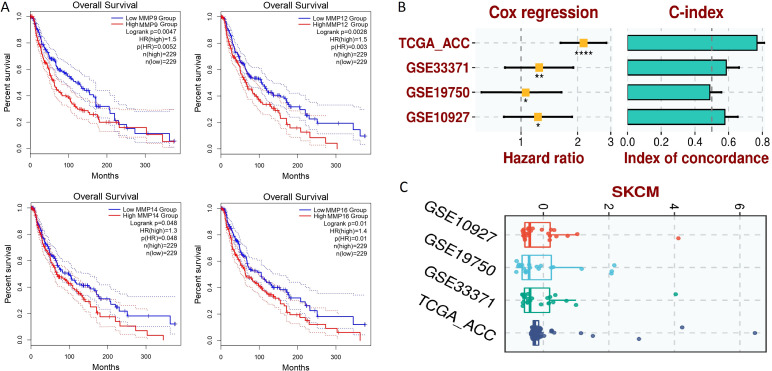
Survival analysis and the construction of the hub gene-based prognostic model. **(A)** GEPIA-based OS analysis of the hub genes in TCGA SKCM samples, **(B)** Univariate Cox regression analysis, **(C)** Risk scores. A p-value < 0.05 was considered significant. p*-value < 0.05; p**-value < 0.01; P****-value < 0.0001.

The prognostic significance of MMP9, MMP12, MMP14, and MMP16 expression in SKCM patients was assessed via the KM Plotter tool. Elevated expression levels of MMP9, MMP12, MMP14, and MMP16 were strongly linked to poorer OS in SKCM patients (**Figure 8A**).

For the analysis of the prognostic model based on MMP9, MMP12, MMP14, and MMP16 genes, this study employed a comprehensive approach utilizing both training and validation datasets. The TCGA-ACC dataset was utilized as the training dataset, providing a foundation for model construction, while the GSE33371, GSE19750, and GSE10927 datasets served as validation datasets to assess the generalizability and robustness of the model. To construct the prognostic model, a stepwise Cox regression model was implemented, incorporating key parameters such as hazard ratio, c-index, and risk score. This iterative approach allowed for the selection of the most informative variables and the optimization of the model’s predictive performance. Through comprehensive evaluation using the c-index, it was determined that the constructed prognostic model effectively and robustly assessed the prognosis of SKCM patients across all analyzed datasets. This finding underscores the utility and reliability of the model in predicting patient outcomes and informing clinical decision-making. **Figures 8B, C** in the study illustrates the performance of the prognostic model across different datasets, providing visual confirmation of its predictive efficacy and demonstrating its potential utility in clinical practice.

### Gene enrichment analysis

**Figure 9:**
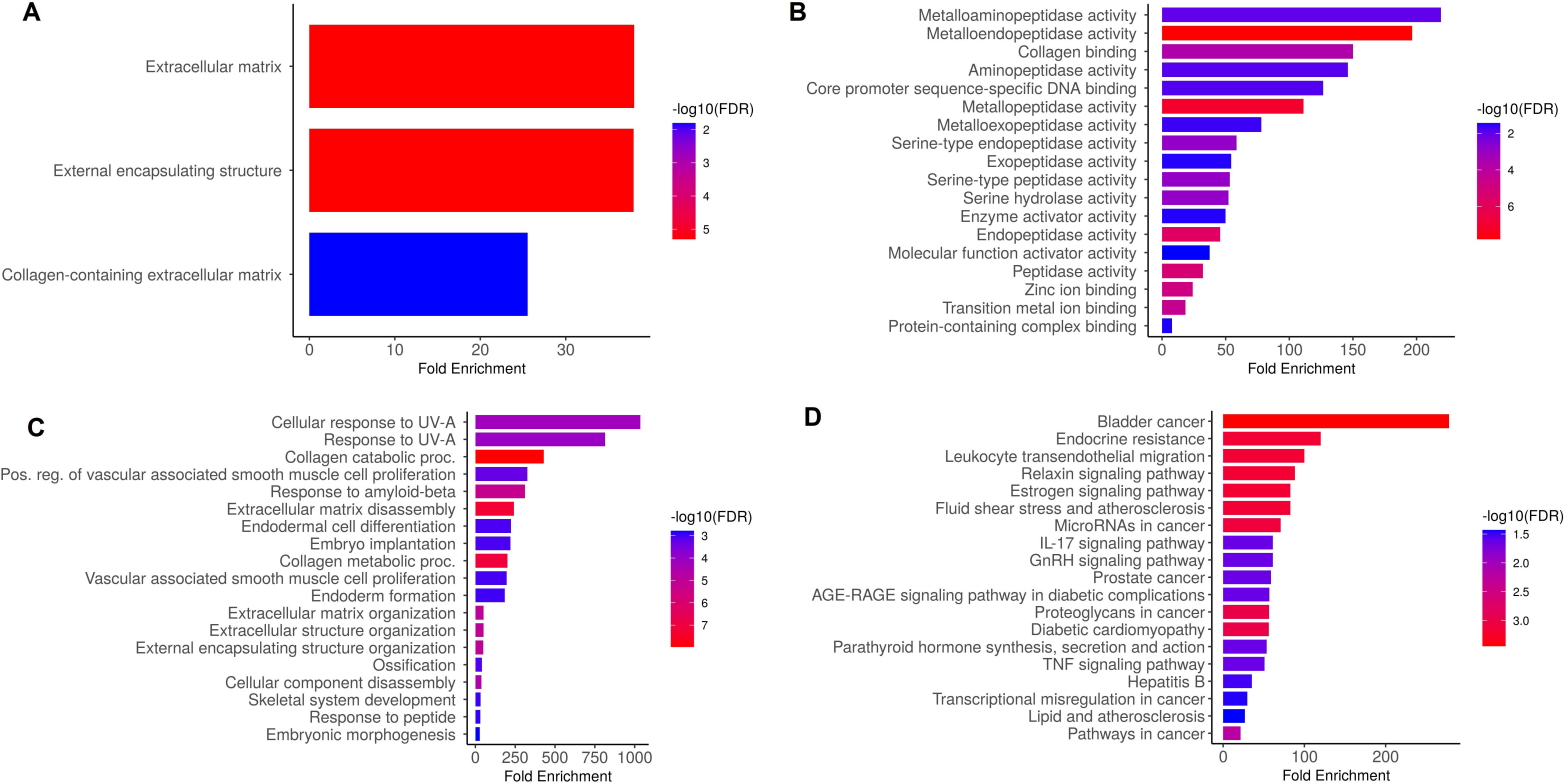
Gene enrichment analysis of MMP9, MMP12, MMP14, and MMP16 via DAVID tool. **(A)** MMP9, MMP12, MMP14, and MMP16 gene-associated CC terms, **(B)** MMP9, MMP12, MMP14, and MMP16 gene-associated BP terms, **(C)** MMP9, MMP12, MMP14, and MMP16 gene-associated MF terms, and **(D)** MMP9, MMP12, MMP14, and MMP16 gene-associated KEGG terms. A p-value < 0.05 was considered significant.

Hub genes were analyzed to figure out their GO and KEGG pathways in SKCM. In the CC, “Extracellular matrix, external encapsulating structure, and collagen-containing extracellular matrix” etc., terms were significantly associated with the MMP9, MMP12, MMP14, and MMP16 (**Figure 9A**). Concerning MF, the “Metalloaminopeptidase activity, Metallendopeptidase activity, and collagen binding” etc., terms were closely associated with the MMP9, MMP12, MMP14, and MMP16 (**Figure 9B**). In BP, some vital functions including “Cellular response to UV-A, response to UV-A, and collagen catabolic proc” etc., terms were significantly associated with the MMP9, MMP12, MMP14, and MMP16 (**Figure 9C**). Moreover, MMP9, MMP12, MMP14, and MMP16-enriched KEGG pathways include “Bladder cancer, endocrine resistance, and relaxin signaling pathway” etc., (**Figure 9D**).

### Cell counting Kit-8 and colony formation assays

**Figure 10:**
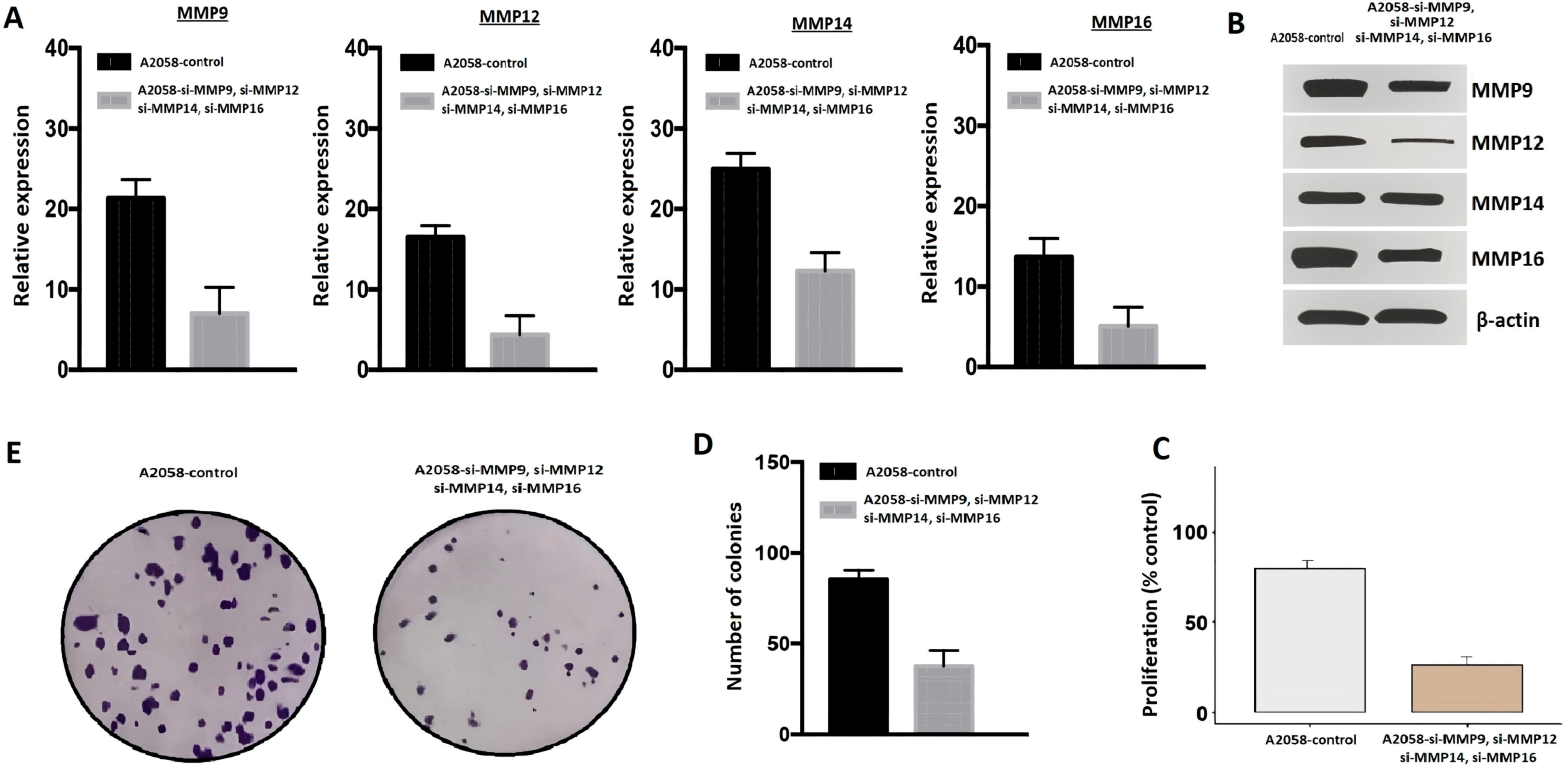
Knockdown of MMP9, MMP12, MMP14, and MMP16 impairs the growth and metastatic potential of A2058 cells. **(A)** The transfection efficiency of si-MMP9, si-MMP12, si-MMP14, and si-MMP16 was checked with the help of RT-qPCR, **(B)** The transfection efficiency of si-MMP9, si-MMP12, si-MMP14, and si-MMP16 was checked with the help of western blot, **(C)** A2058 control and transfected cells were analyzed proliferation, **(D, E)** Colony formation. A p-value < 0.05 was considered significant.

The MMP9, MMP12, MMP14, and MMP16 genes work synergistically to regulate processes such as tissue remodeling, wound healing, and cancer invasion. Therefore, the simultaneous silencing of MMP9, MMP12, MMP14, and MMP16, and was carried out in A2058 cells using siRNA to analyze their functional synergetic impact on the different parameters. The RT-qPCR and western blot analysis results, as depicted in **Figures 10A, B**, unequivocally demonstrated a significant reduction in the mRNA and protein expression levels of MMP9, MMP12, MMP14, and MMP16 in the transfected A2058 cells in comparison to the control A2058 cells. To gain deeper insights into the repercussions of MMP9, MMP12, MMP14, and MMP16 knockdown, the conducted CCK-8 and colony-forming assays, providing compelling evidence of decreased cellular proliferation in the cells with silenced MMP9, MMP12, MMP14, and MMP16, in contrast to the control A2058 cells (**Figures 10C–E**).

### Drug prediction and molecular docking analysis

DrugBank database was searched to explore potential drugs that could down-regulate the expression of MMP9, MMP12, MMP14, and MMP16 genes in the context of SKCM treatment. The findings unveiled two promising drugs (Estradiol and Calcitriol) within this database that exhibit the potential to reduce the expression of MMP9, MMP12, MMP14, and MMP16 genes.

**Figure 11:**
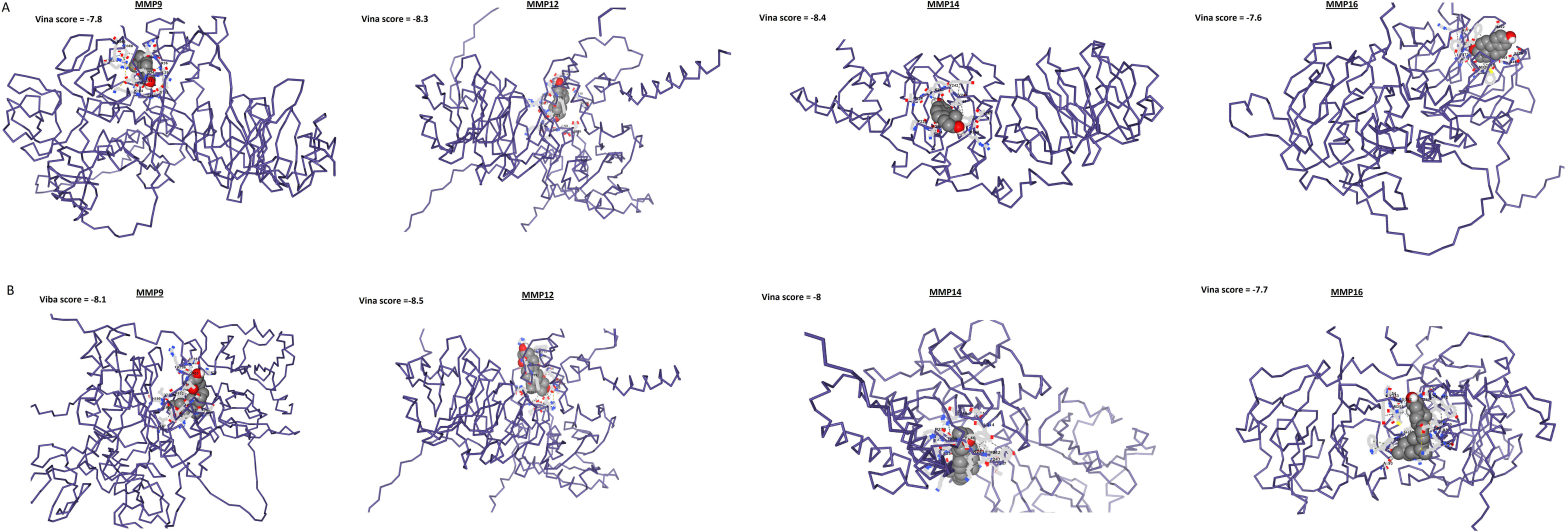
Molecular docking outcomes of Estradiol and Calcitriol with MMP9, MMP12, MMP14, and MMP16 hub genes. The MMP9, MMP12, MMP14, and MMP16 proteins are represented in blue structures, while Estradiol and Calcitriol drugs are depicted in gray molecules, showcasing their docking interactions with the target proteins.

In the next step, the role of Estradiol and Calcitriol in the expression reduction was further validated through molecular docking analysis. Docking results show that binding affinities of Estradiol and Calcitriol with MMP9, MMP12, MMP14, and MMP16 vary between -7.7 and -8.5 kcal/mol (**Figure 11**). The binding affinities of -7.7 to -8.5 kcal/mol suggest a relatively strong interaction between Estradiol and Calcitriol with MMP9, MMP12, MMP14, and MMP16 proteins (**Figure 11**). In summary, while the binding affinities suggest strong interactions, the actual effectiveness of Estradiol and Calcitriol as inhibitors for MMP9, MMP12, MMP14, and MMP16 proteins would require comprehensive *in vitro* and *in vivo* studies.

## Discussion

SKCM is a malignant neoplasm originating from melanocytes, the pigment-producing cells found in the skin (37). It is the most lethal form of skin cancer due to its propensity for metastasis, and its incidence has been steadily increasing in recent years, making it a significant public health concern (38, 39). The incidence of SKCM varies geographically, with higher rates observed in regions with greater sun exposure (40). Fair-skinned individuals and those with a history of intense sun exposure or sunburns are at a higher risk of developing SKCM (41, 42). Moreover, a family history of melanoma and certain genetic factors can also increase the chances of SKCM development (43, 44). Recently, numerous preceding studies have emphasized the role of MMPs in the development and progression of cancer (45–47). Additionally, MMPs play a significant role in immune evasion (48). They can modulate the tumor microenvironment by cleaving ECM components, which can alter the recruitment and activation of immune cells (49). For instance, certain MMPs have been shown to facilitate the infiltration of immunosuppressive cells, such as regulatory T cells and myeloid-derived suppressor cells, into the tumor site (50). This immune modulation not only helps tumors evade detection by the immune system but also supports their growth and survival (51). Considering the pivotal function of ECM alteration in the advancement of tumors, gaining insights into the contribution of particular MMPs to SKCM could offer valuable information regarding its underlying mechanisms.

Out of the total analyzed 24 MMP family members, MMP9, MMP12, MMP14, and MMP16 genes were recognized as the key genes due to high centrality among others. Expression analysis indicated that MMP9, MMP12, MMP14, and MMP16 genes were significantly up-regulated at both mRNA and protein levels in SKCM. Therefore, it is speculated that elevated levels of MMP9, MMP12, MMP14, and MMP16 may interfere with the wound healing process in SKCM. These observations align with previous studies that have reported elevated expression of specific MMPs in various cancer types. For instance, earlier studies (52, 53) demonstrated the overexpression of MMP9, MMP12, MMP14, and MMP16 in breast and kidney cancers, emphasizing their role in tumor progression and metastasis. However, to our knowledge, this study is the first to report overexpression of these key genes in SKCM.

The inverse correlation between promoter methylation and gene expression observed in this study is in concordance with established epigenetic mechanisms of gene regulation (54, 55). Previous research in SKCM and other cancer types has emphasized the significance of promoter methylation in gene expression control (56, 57). A study by Huang et al. (58) in SKCM highlighted the hypomethylation of genes associated with cancer progression, consistent with the findings of this study regarding the MMP family.

ROC analysis demonstrated the diagnostic potential of MMP9, MMP12, MMP14, and MMP16 in SKCM. These findings align with earlier studies emphasizing the diagnostic and prognostic utility of MMPs in cancer. Previous studies investigated the diagnostic value of MMPs in various cancers, including melanoma, reinforcing the significance of these genes as potential biomarkers (59–61). Moreover, the present study identified a low mutation rate in the MMP family genes within SKCM. This finding resonates with earlier research suggesting that genetic mutations in MMPs may not be frequent drivers of cancer development (62). Previous studies have also reported relatively low mutation rates in MMPs, emphasizing the complex regulation of MMP gene expression (63, 64). However, it is essential to acknowledge that genetic mutations may have context-specific roles in cancer biology (65–68), and additional investigations are required to fully comprehend their impact.

The prognostic value of high expression levels of MMP9, MMP12, MMP14, and MMP16 in various cancers, as indicated in this study, has also been reported in prior research. For example, a study by McGowan et al. (69) demonstrated a link between elevated MMP expression and adverse clinical outcomes in breast cancer patients. However, to our knowledge, this study is the first to report prognostic values of MMP9, MMP12, MMP14, and MMP16 in SKCM.

In our quest to identify promising therapeutic drugs for the treatment of SKCM, Estradiol and Calcitriol drugs were selected from the DrugBank database after a thorough examination of their pharmacological properties and their known effects on dysregulated MMP9, MMP12, MMP14, and MMP16 genes. Estradiol, a potent estrogen hormone, and Calcitriol, the active form of vitamin D, have garnered significant interest in the realm of cancer research due to their ability to modulate gene expression patterns (70–72). Specifically, previous studies have highlighted their potential in regulating the activity of Matrix Metalloproteinases (MMPs), which play pivotal roles in cancer progression and metastasis (73, 74). By targeting MMP activity, it is possible to impede these processes and potentially limit the spread of cancer. Importantly, the efficacy of Estradiol and Calcitriol in modulating MMP activity has been demonstrated across different cancer types, providing a compelling basis for their consideration in the context of SKCM treatment (70–72). Studies have shown that these compounds can regulate the expression and activity of MMPs, thereby exerting anti-tumor effects and inhibiting metastatic spread. By targeting MMP9, MMP12, MMP14, and MMP16, it is conceivable that Estradiol and Calcitriol could disrupt critical pathways involved in melanoma progression, offering a novel and potentially effective approach for combating this aggressive form of skin cancer. Looking ahead, future *in vitro* validations could involve treating SKCM cell lines with Estradiol and Calcitriol to assess changes in MMP expression and activity, as well as evaluating effects on cell proliferation, migration, and invasion. Additionally, *in vivo* studies using relevant animal models of SKCM could further elucidate the therapeutic potential of these compounds by examining their impact on tumor growth, metastasis, and overall survival. Together, these experimental approaches would provide critical insights into the effectiveness of Estradiol and Calcitriol as therapeutic agents, potentially leading to their integration into clinical treatment strategies for patients with SKCM.

Research on MMPs in cancer is continually evolving, and numerous complexities in their roles and regulation have been recognized. While this study provides valuable insights, further in-depth exploration of the regulatory mechanisms governing MMP expression is warranted for a comprehensive understanding of their diagnostic, prognostic, and therapeutic potential.

The study’s strengths include its thorough analysis of 24 MMP genes and its use of a multi-omics approach, integrating protein-protein interaction networks, gene expression profiling, and functional assays. This comprehensive methodology and the use of advanced tools and databases ensure robust findings and potential therapeutic insights. However, the study’s limitations include the use of a limited number of cell lines, which may not fully represent SKCM’s clinical diversity, and the reliance on single-method validation for some analyses. These factors suggest a need for further research to confirm and expand upon the study’s results.

To build on the findings of this study, future research should incorporate a larger and more diverse panel of SKCM cell lines, as well as primary tumor samples, to better capture the clinical heterogeneity of the disease. Additionally, incorporating *in vivo* models could enhance the relevance of the results and validate the therapeutic potential of targeting specific MMPs. Expanding the analysis to include other omics layers, such as epigenomics and metabolomics, would provide a more holistic understanding of the molecular mechanisms driving melanoma progression. Lastly, integrating CRISPR-Cas9 gene editing or RNA interference techniques could be used to validate key MMP targets in functional assays and uncover their role in SKCM more definitively.

## Conclusion

Our study highlights MMP9, MMP12, MMP14, and MMP16 as critical hub genes in SKCM, showing elevated mRNA and protein levels compared to normal controls. Their reduced promoter methylation suggests hypomethylation contributes to their overexpression. These genes are rarely mutated, indicating that their dysregulation is likely due to expression changes rather than genetic mutations. Elevated expression correlates with poorer survival and a prognostic model incorporating these genes accurately predicts patient outcomes. Functional assays reveal that silencing these genes impairs cellular proliferation. Drug prediction and molecular docking suggest Estradiol and Calcitriol as potential inhibitors, though further studies are needed. These findings underscore the genes’ roles as biomarkers and therapeutic targets in SKCM.

## Data Availability

The original contributions presented in the study are included in the article/**Supplementary Material**. Further inquiries can be directed to the corresponding authors.
