# Supplementary figures and images for: Identification and validation of matrix metalloproteinase hub genes as potential biomarkers for Skin Cutaneous Melanoma
Source: Front Oncol. 2024 Oct 18;14:1471267. doi: 10.3389/fonc.2024.1471267 (PMC11527786; doi:10.3389/fonc.2024.1471267)

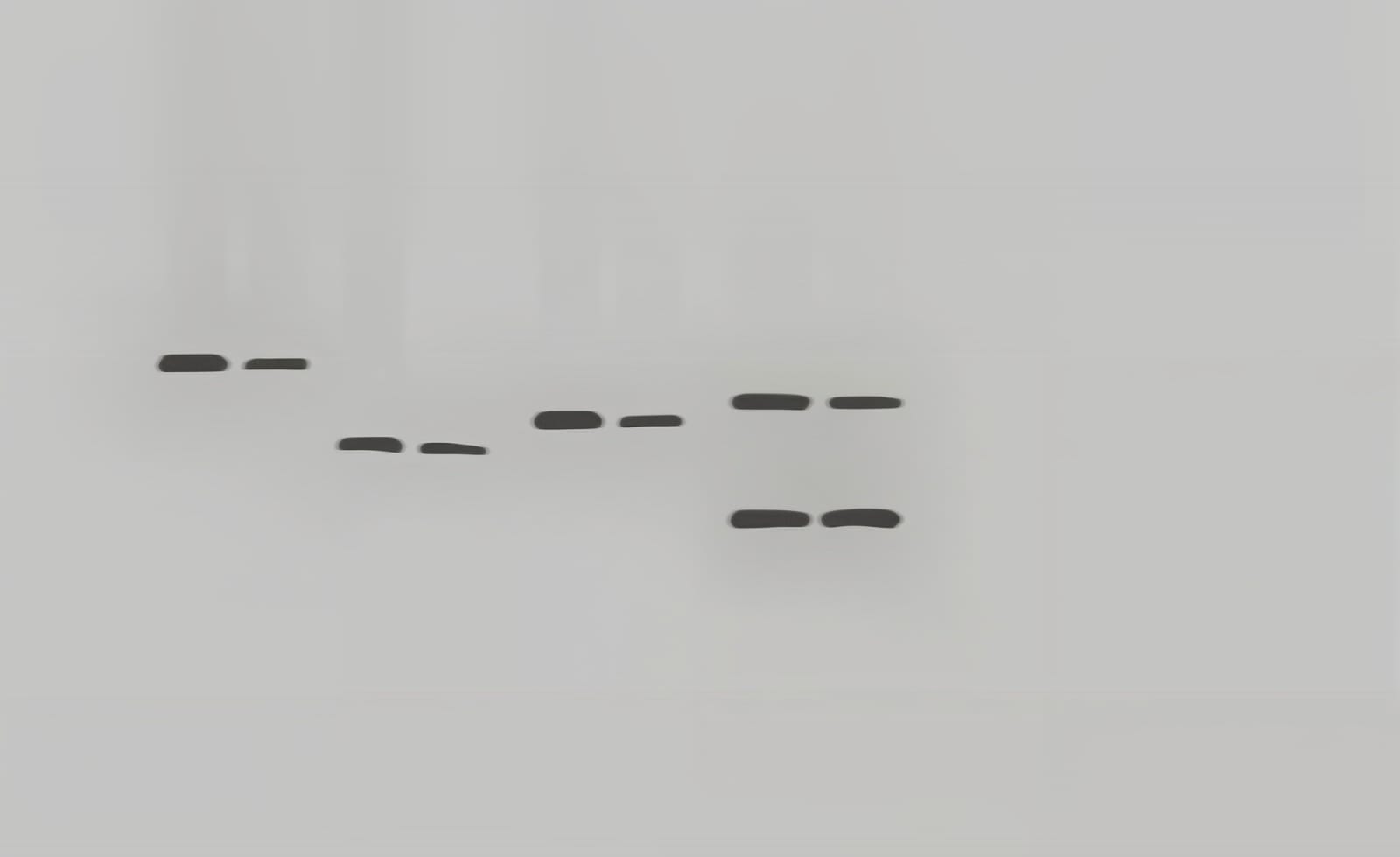

Supplement: Supplementary file 1 [file Image1.jpeg]

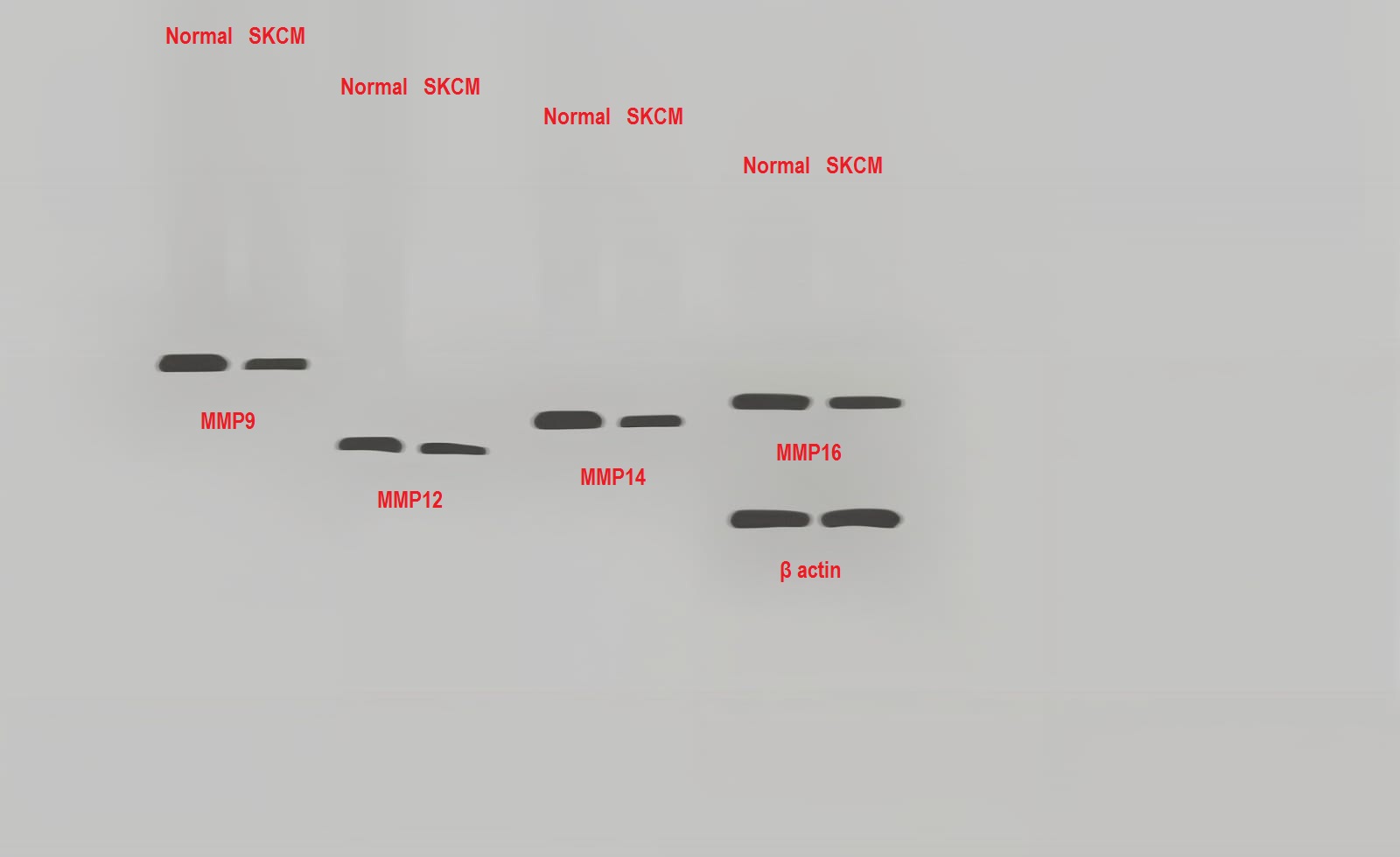

Supplement: Supplementary file 2 [file Image2.jpeg]

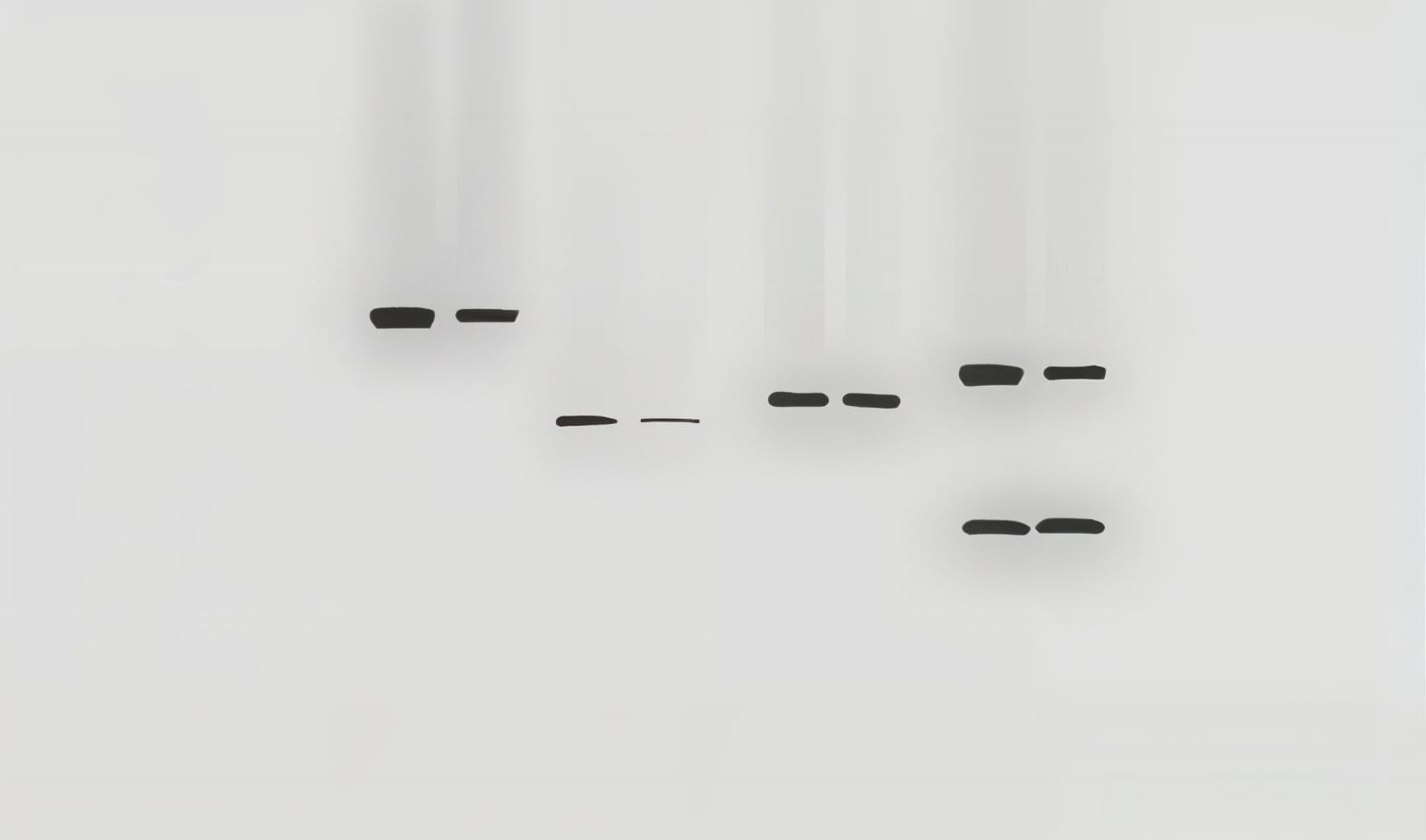

Supplement: Supplementary file 3 [file Image3.jpeg]

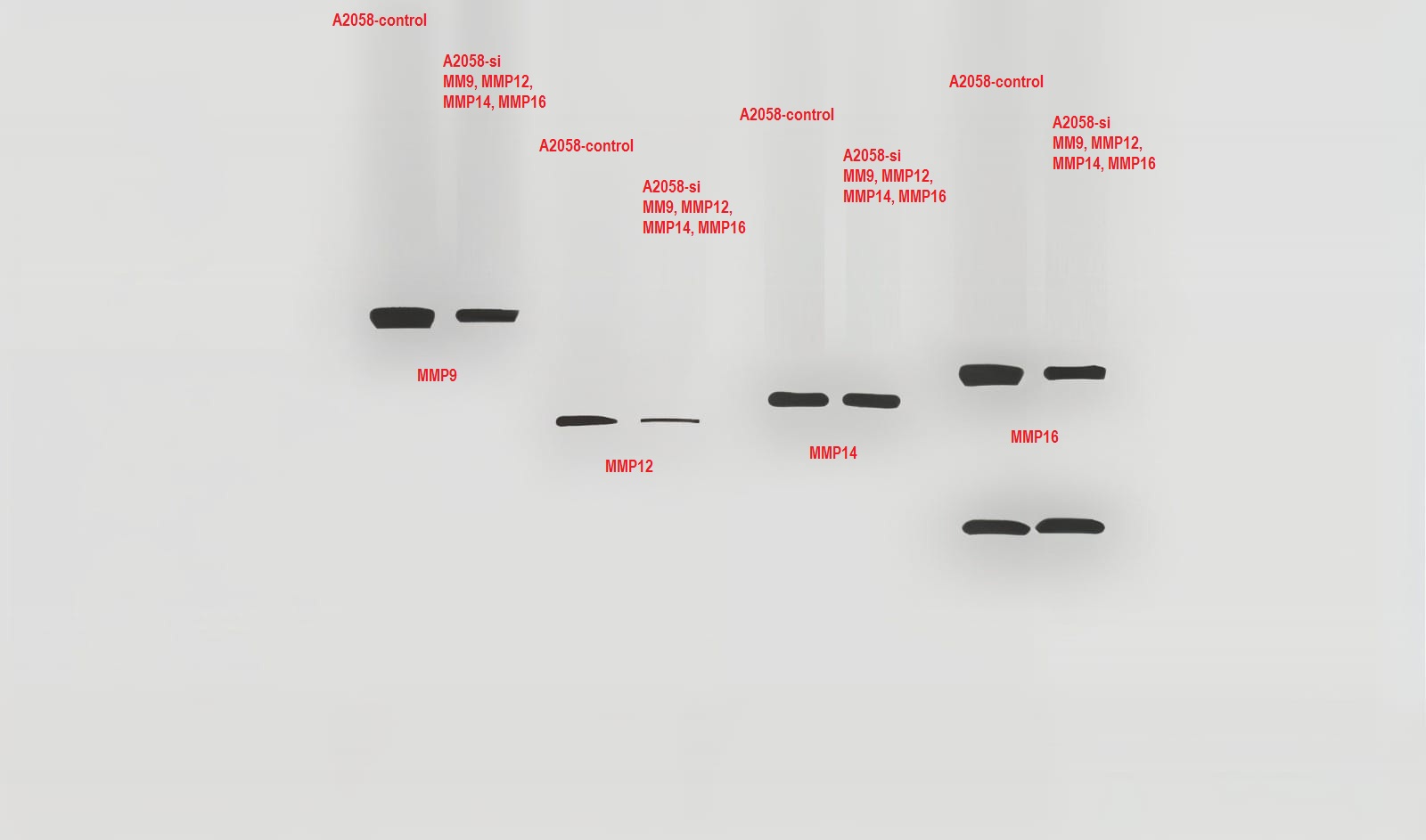

Supplement: Supplementary file 4 [file Image4.jpeg]
